# Atrial Fibrillation Specific Exercise Rehabilitation: Are We There Yet?

**DOI:** 10.3390/jpm12040610

**Published:** 2022-04-10

**Authors:** Benjamin J. R. Buckley, Signe S. Risom, Maxime Boidin, Gregory Y. H. Lip, Dick H. J. Thijssen

**Affiliations:** 1 Liverpool Centre for Cardiovascular Science, University of Liverpool, William Henry Duncan Building, Liverpool L7 8TX, UK; gregory.lip@liverpool.ac.uk; 2 Cardiovascular and Metabolic Medicine, Institute of Life Course and Medical Sciences, University of Liverpool, Liverpool L7 8TX, UK; 3 Department of Cardiology, Herlev and Gentofte University Hospital, 2730 Herlev, Denmark; signe.stelling.risom@regionh.dk; 4 Institute of Nursing and Nutrition, University College Copenhagen, 1799 Copenhagen, Denmark; 5 Department of Clinical Medicine, Faculty of Health and Medical Sciences, University of Copenhagen, 1799 Copenhagen, Denmark; 6 Liverpool Centre for Cardiovascular Science, Liverpool John Moores University, Liverpool L3 3AF, UK; m.boidin@ljmu.ac.uk (M.B.); dick.thijssen@radboudumc.nl (D.H.J.T.); 7 Department of Physiology, Research Institute for Health Science, Radboud University Medical Centerum, P.O. Box 9101, 6500 HB Nijmegen, The Netherlands

**Keywords:** rehabilitation medicine, physical activity, exercise, atrial fibrillation, cardiovascular disease, preventive cardiology, vascular health, atrial health, secondary prevention

## Abstract

Regular physical activity and exercise training are integral for the secondary prevention of cardiovascular disease. Despite recent advances in more holistic care pathways for people with atrial fibrillation (AF), exercise rehabilitation is not provided as part of routine care. The most recent European Society of Cardiology report for AF management states that patients should be encouraged to undertake moderate-intensity exercise and remain physically active to prevent AF incidence or recurrence. The aim of this review was to collate data from primary trials identified in three systematic reviews and recent real-world cohort studies to propose an AF-specific exercise rehabilitation guideline. Collating data from 21 studies, we propose that 360–720 metabolic equivalent (MET)-minutes/week, corresponding to ~60–120 min of exercise per week at moderate-to-vigorous intensity, could be an evidence-based recommendation for patients with AF to improve AF-specific outcomes, quality of life, and possibly prevent long-term major adverse cardiovascular events. Furthermore, non-traditional, low-moderate intensity exercise, such as Yoga, seems to have promising benefits on patient quality of life and possibly physical capacity and should, therefore, be considered in a personalised rehabilitation programme. Finally, we discuss the interesting concepts of short-term exercise-induced cardioprotection and ‘none-response’ to exercise training with reference to AF rehabilitation.

## 1. Why Do We Need AF-Specific, Exercise-Based Rehabilitation?

Regular physical activity and exercise training are integral for the secondary prevention of cardiovascular disease (CVD), as demonstrated in both interventional and real-world studies [1,2,3,4]. However, 42% of general western populations do not meet the recommended physical activity guidelines (i.e., 150 min of moderate or 75 min of vigorous intensity physical activity/week) [5]. Further, those with CVD are typically less active than the general population yet stand to benefit the most from exercise training [6]. For example, Jeong et al. [6] demonstrated that, in 131,558 individuals with CVD, every 500 metabolic equivalent (MET)-minute/week increase in physical activity resulted in a 14% risk reduction in mortality, whereas in 310,240 participants without CVD, risk reduction was only 7%. Interestingly, while individuals without CVD benefited the most between 1 and 500 METs-min/week of physical activity, the benefit in those with CVD continued above 500−1000 METs-min/week. Thus, exercise interventions, such as cardiac rehabilitation, are an essential component of secondary and tertiary cardiovascular disease management. It is also apparent that for rehabilitation programmes, one size does not fit all [7], and optimised interventions should be developed to enhance outcomes for different population groups.

There is increasing interest for a more personalised approach to exercise-based cardiovascular rehabilitation, especially since cardiac (chronotropic incompetence, diastolic dysfunction, and systolic dysfunction), non-cardiac (vascular function/structure, skeletal myopathy, and autonomic control), comorbidities (ageing, metabolic diseases, and cardiovascular diseases), and external (exercise adherence/dose/intensity) factors influence individual responses [8]. In particular, these factors have been shown to moderate baseline and even the relative change in cardiorespiratory fitness (V˙O_2_-peak) following exercise training [8].

From the cardiac and comorbidity perspective, there are specific exercise guidelines for a number of cardiovascular conditions, including ischaemic heart disease, heart failure, hypertension, and peripheral artery disease [9,10]. These condition-specific guidelines help to personalise exercise prescription at a group level and represent an initial step towards the optimisation of individual patient benefit.

The most recent European Society of Cardiology (ESC) guidelines for the diagnosis and management of atrial fibrillation (AF) repeatedly highlight the importance of cardiopulmonary exercise testing (CPET) and cardiorespiratory fitness, as well as stating that ‘*patients should be encouraged to undertake moderate-intensity exercise and remain physically active to prevent AF incidence or recurrence’* [11]. Furthermore, the latest ESC Guidelines on Sports Cardiology and Exercise in Patients with Cardiovascular Disease emphasise the primary and secondary preventive effects of physical activity and present specific guidelines for healthy individuals and those with cardiovascular disease risk factors [9]. Specific secondary preventive exercise guidelines are, however, not provided for people with AF.

Exercise rehabilitation presents an important and potentially impactful initiative for people with AF and the health services they use. However, more specific exercise-rehabilitation guidelines are needed for people with AF. We, therefore, conducted a narrative review of the literature, supported by recent systematic reviews and primary studies, to produce more nuanced exercise rehabilitation thresholds for people with AF. By working towards such an objective, we can better guide both healthcare professionals and patients by recommending AF-specific exercise-based rehabilitation and/or guide future research to help contribute to this research gap.

## 2. Methods

Original trials were included from a Cochrane systematic review of exercise-based cardiac rehabilitation [12] and three more recent systematic reviews investigating exercise-based cardiac rehabilitation [13,14] and different types of exercise interventions [15] for adults with AF. In addition, we included recent and relevant prospective and retrospective studies that investigated the impact of exercise or physical activity on health outcomes for people with AF.

It is well known that there is a seemingly counterintuitive increased risk of AF for some who engage in excessive amounts of vigorous-intensity exercise (i.e., ‘athletic AF’) [16]. However, athletic AF is only observed following exceptionally high levels of vigorous endurance training, such as >5000 MET-min/week or 5 to 10-fold the existing physical activity guidelines [16]. Given that athletic AF represents a relatively small percentage of the AF population, the present review will focus on the impact of physical activity and exercise training on the secondary prevention of AF and associated outcomes in non-athletic populations only.

**Figure 1:**
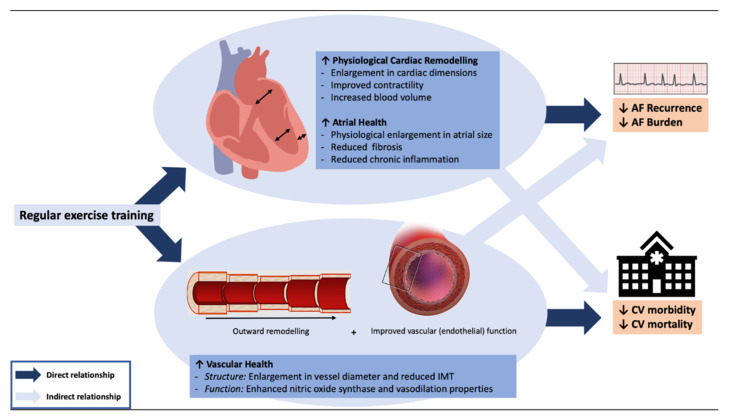
**Proposed mechanisms explaining the long-term AF protection from regular exercise training.** We propose atrial health has a direct impact on AF recurrence/burden and an indirect impact on AF morbidity whereas vascular health has a direct impact on AF morbidity and an indirect impact on AF recurrence/burden. Dark blue arrows represent a direct relationship. Light blue arrows represent an indirect relationship.

Data from relevant and eligible primary studies were extracted by one reviewer to define the AF population, exercise intervention or physical activity behaviour details (i.e., frequency, intensity, time, and type (FITT) principles), follow-up time points, and subsequent health outcomes, stratified by study design (Table 1). All extracted data were quality checked for accuracy by a second reviewer. Given the heterogeneity in investigated outcome measures across the literature, we decided to focus on a priori serious adverse events (SAE; mortality, hospitalization, and stroke), physical capacity (including the 6 min walk test and V˙O_2_-peak), AF specific outcomes (including recurrence and/or time in AF), and quality of life (QoL). These data were then used to determine if more nuanced exercise recommendations could be proposed and highlight where further research is needed. Aligned with the extracted data regarding AF-specific exercise rehabilitation, we then discuss the hypothesised mechanisms of long-term (Figure 1) and possible acute AF protection following exercise training and physical activity. Finally, we conclude with key take-home messages and recommendations for future research in this area.

## 3. Evidence Informing AF-Specific Rehabilitation

### 3.1. Study Characteristics

Table 1 summarises the reviewed primary research and available data of interest extracted from each study. Of the included 21 studies (22 publications), 13 were randomised controlled trials and 8 were pre-post or cohort studies. Sample size ranged between 66,692 [19] and 18 [20], and female representation ranged between 77% [18] and 12% [21], with 11 studies investigating paroxysmal and/or persistent AF subtypes, [18,22,23,24,25,26,27,28,29,30,31], 4 permanent AF, [21,32,33,34], 2 all-AF subtypes [2,35], and 4 studies did not stratify their results by AF subtype [17,19,36,37]. Included interventions were moderate-intensity continuous training, high-intensity interval training, cardiac rehabilitation (moderate-vigorous intensity continuous training and resistance training), low-to-moderate continuous training (Yoga, Qigong), inspiratory muscle training, and resistance plus moderate-intensity continuous training (circuit training). Follow-up for outcomes of interest (SAE, physical capacity, AF-specific outcomes, and quality of life) ranged between 8-weeks [21] and 9-years [35].

### 3.2. Intervention/Cohort Details and Long-Term Outcomes

Population-based studies. In a cohort study of 66,692 participants with newly diagnosed AF, performing > 30 min of moderate or >20 min of vigorous intensity exercise at least once per week was associated with significantly lower risk of heart failure and mortality (and a 10–14% lower risk of ischaemic stroke, although the latter was not significant) [19]. In subgroup analyses of patients with AF who went from previously inactive to active, an energy expenditure of 1000–1499 MET-minutes/week, corresponding to 170–240 min per week of moderate intensity exercise, was consistently associated with a lower risk of mortality, stroke, and heart failure in patients with AF [19]. In another cohort study of 1117 patients with AF, participants were stratified by activity level (i.e., inactive, not meeting physical activity guidelines, and meeting physical activity guidelines). The authors reported that meeting the 150 min of moderate-intensity physical activity guidelines was associated with lower risk of all-cause mortality, cardiovascular-related mortality, cardiovascular morbidity, and stroke compared to those not meeting the guidelines and those who were inactive [35]. This work highlights the potential potency of meeting (150 min of moderate intensity physical activity/week) and exceeding the general physical activity guidelines (170–240 min of moderate intensity physical activity/week) on long-term clinical outcomes in patients with AF. However, as these data are from cohort studies, the findings are associations, not causal.

Intervention studies. The most researched exercise-based intervention included in this review was cardiac rehabilitation with seven included studies across four RCTs [22,24,28,29] and three cohort studies [2,30,37]. There were consistent benefits shown in studies that investigated comprehensive cardiac rehabilitation or an intervention designed to reflect cardiac rehabilitation. The various types of intervention included an exercise programme consisting of 1–3 sessions/week of 30–60 min moderate-to-vigorous intensity exercise for 9-weeks to 6-months. A variety of benefits was observed across all studies including fewer major adverse cardiovascular events such as mortality, hospitalisation, and stroke; [2] less progression of AF subtype from paroxysmal to sustained AF; [30] lower AF recurrence; [22] higher physical capacity; [24,28,29,37] and better quality of life [24,28].

A total of four included studies investigated less traditional low-moderate intensity continuous exercise programmes including Yoga, [31] Mediyoga (a therapeutic form of meditative yoga based on deep breathing) [26,27], and Qigong (slow and graceful movements with a focus on breathing) [33]. In a pre-post study, Lakkireddy et al. showed that following 12-weeks of Yoga twice a week, episodes of AF were significantly reduced and quality of life significantly improved (including Physical Functioning, General Health, Vitality, Social Functioning, and Mental Health subscales). More recently, Wahlström et al. demonstrated that 12 weeks of Mediyoga once per week resulted in significantly increased physical capacity (measured via 6MWT) and the mental component of quality of life compared to a control [27]. In a later study, Mediyoga was shown to increase within arm improvements in bodily pain, general health, social function, mental health, and mental component summary scores (as measured by the Short-Form Health Survey) within the MediYoga group, but no significant differences were seen when compared to an active control or usual care [26]. Finally, two 90 min sessions of Qigong for 16-weeks demonstrated increased physical capacity compared to controls (measured via the 6MWT) [33]. This work provides promise for alternative low-moderate intensity continuous exercise, such as Yoga, for improved quality of life and even physical capacity for people with AF. No known study has yet explored whether these benefits also translate to clinical or AF-specific outcomes following this type of exercise.

Three studies investigated the effect of vigorous-intensity exercise for patients with AF. A programme of three sessions per week of vigorous-intensity exercise for 12-weeks was shown to significantly improve time in AF (reduced AF burden), improve physical capacity, and improve quality of life compared to usual care across two RCTs [18,32]. However, in one post hoc analysis from an RCT, two sessions of high-intensity interval exercise per week for 6-months improved quality of life and V˙O_2_-peak in both intervention and usual care groups (no significant difference between groups). However, the sample of patients in this study underwent cardiac resynchronisation therapy and represented only 18 participants with AF across both groups [20]. One RCT investigated low- vs high-intensity exercise consisting of two sessions of interval and circuit-based training twice per week for 12-weeks [25]. Both groups demonstrated an improvement in V˙O_2_-peak but no group-time interaction was observed. Furthermore, there was no difference in AF burden (measured as ECGs with AF/ECGs without AF) between the two exercise groups. Although the measurement of AF burden with non-continuous methods (as conducted in the latter study) has its limitations [38], this work highlights a topical argument regarding whether we should focus on exercise duration or exercise intensity.

### 3.3. Should We Promote Increased Intensity or Duration of Exercise for Improved Outcomes?

By observing data from other more intensively researched cardiovascular conditions [39,40], it is possible that the overall energy expenditure is the most important variable for optimal patient outcomes. In line with this hypothesis, two systematic reviews with meta-analyses and meta-regression have shown that total energy expenditure of an overall exercise programme was the strongest predictor of improvement in exercise capacity for patients with heart failure [39,40]. It was concluded that at least 460 kcal of weekly energy expenditure may elicit the greatest changes in cardiorespiratory fitness for patients with heart failure (regardless of how those kcals are achieved, i.e., high-intensity interval training vs. moderate-intensity continuous training) [39]. Similarly, in a multi-centre comparison, Williams et al. [41] found that in both healthy populations and participants with cardiovascular disease, higher amounts and intensity of exercise increased the likelihood of cardiorespiratory fitness improvement. Furthermore, it has been shown (in a healthy sample) that individual cardiorespiratory ‘non-response’ to exercise training is abolished by increasing the volume of aerobic continuous exercise [42]. Therefore, it seems that by increasing the overall exercise dose, whether by increasing the volume and/or intensity of an exercise programme, more individuals (even those most likely not to respond) have improved odds of benefiting from the programme. The overall dose of an exercise programme is a product of the frequency of sessions, intensity, and time (duration of individual sessions and programme). All of these can be manipulated to personalize the exercise programme.

By taking an overall exercise dose approach, a *minimum* exercise threshold of 360–720 MET-minutes/week would correspond to 60–120 min of exercise training per week at moderate-to-vigorous intensity (Graphical Abstract). This proposed threshold is typical of a cardiac rehabilitation programme and the majority of aerobic-type exercise interventions included within this review, which have demonstrated clinical benefit. Nonetheless, exceeding this exercise dose would increase one’s likelihood of benefit, whether by increasing the number of sessions per week or the intensity of exercise in an individual session. For example, in a rigorous between and within study design, 78 healthy participants were divided into five groups comprising 1–5 60 min exercise sessions for 6-weeks. Non-responders in V˙O_2_-peak at 6-weeks were prescribed an additional two sessions per week. Findings showed that that in groups 1, 2, 3, 4, and 5, 69%, 40%, 29%, 0%, and 0% of individuals, respectively, were non-responders. Interestingly, after increasing the exercise programme by an additional two sessions for all non-responders across all groups, all non-response in V˙O_2_-peak was eliminated [42]. This highlights the potency of increasing the dose of an exercise programme via increased exercise volume. However, increasing intensity and duration of exercise may enhance the befits from exercise further. Using a multi-centre comparison of V˙O_2_-peak trainability between high- and low-volume interval training and moderate-intensity continuous training, with a total of 677 participants representing 18 different intervention studies, high-volume and high-intensity interval training had more responders in V˙O_2_-peak improvement compared to low-volume and high-intensity interval training and moderate intensity continuous training [41]. Thus, gradually increasing both intensity and duration of exercise may be the optimal strategy for reducing ‘non-response’ in clinical exercise programmes. This concept could be titrated on an individualised basis for those who may not respond to the initial dose. This may explain the previously discussed findings that increasing one’s activity level up to three-fold the current exercise guidelines (1000–1499 MET-minutes/week) had the strongest association with improved major adverse cardiovascular outcomes at 2-year follow-up in previously inactive patients with AF [19]. However, this was based on self-reported data, which can vary considerably from device-based measurement depending on the method used and population investigated [43]. Therefore, this may represent an elevated threshold for improved long-term prognosis in patients with AF. It is also important to note that there has been no research investigating non-response to exercise training in patients with AF, and this warrants future investigation. This may be particularly important for those with concomitant conditions such as AF and heart failure (especially those with preserved ejection fraction) who may be less responsive to initial training [44,45].

### 3.4. Mechanisms of AF Protection from Long-Term Exercise Training

Although exercise training improves individual risk factors, collectively, only ~50% of the exercise-induced benefit to health can be explained by risk factor improvement [46,47]. There is now a clear body of work that has shown the favourable adaptations seen in vascular structure and function following exercise training [48], which can help to explain this so called “risk factor gap” [49,50,51]. Figure 1 depicts the hypothesised contribution of risk factor modification, specifically physiological cardiac remodelling (enlargement in cardiac dimension, improved contractility, and increased blood volume), atrial health (physiological increase in atrial size with associated reduction in fibrosis and inflammation), and vascular health (increased diameter size, improved vascular function) to explain the benefit of regular exercise training on AF burden and major adverse events. Here, we propose that the beneficial impact of exercise training on physiological cardiac remodelling [52] and an AF substrate [16] has a direct impact on AF burden and recurrence and an indirect impact on AF morbidity. Conversely, we propose that the beneficial impact of exercise training on vascular structure and function [49,53] has a direct impact on AF morbidity and an indirect impact on AF burden and recurrence (Figure 1).

When referring to increased cardiac remodeling and atrial health (particularly increased atrial size), Figure 1 represents *physiological* hypertrophy/cardiac remodelling as a direct result of exercise training. Physiological hypertrophy/cardiac remodelling is associated with enhanced cardiac function whereas *pathological* hypertrophy/cardiac remodelling is associated with increased fibrosis, cardiac dysfunction, and can be arrhythmogenic. Vascular structure typically refers to the thickness of the arterial wall, measured as the intima-media thickness (IMT), and the diameter of the lumen, measured using vascular ultrasound. Vascular function refers to the ability of the artery to vasodilate and/or vasoconstrict dependent on the stimulus used. Commonly used measures of vascular function include flow-mediated dilatation (largely endothelial dependent) and carotid artery reactivity (largely catecholamine dependent) [48].

### 3.5. Is There an Acute, AF-Specific, Cardioprotective Effect of Exercise for People with AF?

In a novel prospective cohort study of 1410 participants with risk factors for AF and stroke, Bonnesen et al. [36] created a dynamic parameter describing within-individual changes in daily physical activity, i.e., average daily physical activity in the last week compared to the previous 100-day average. They showed that a 1 h decrease in daily physical activity during the last week increased the odds of AF onset the next day by 24%. This provides the first known data suggesting a possible acute AF-specific protective effect of regular physical activity. Furthermore, the strongest association was observed in the group with the lowest activity overall, where a 1 h reduction in an individual’s physical activity increased the odds of AF by 60% for the those in the third lowest activity stratum. Interestingly, the most physically active participants were somewhat protected from transient reductions in physical activity with lower odds of AF following acute physical inactivity. Although these findings are exciting, it is important to emphasise that the analysis stems from a sub-study of a larger prospective study and there are limitations with regard to the detail of physical activity data retrieved from implantable loop recorders. Further, there is the possibility of reverse causality, whereby undetected AF or other condition-specific symptoms may have affected physical activity levels prior to an AF event.

The idea of immediate and acute cardioprotection from exercise is not a new concept and has been previously termed cardiovascular preconditioning or short-term exercise-induced cardioprotection, typically researched in patients with atherosclerotic CVD [54,55]. These short-term exercise-induced benefits stem from pre-clinical evidence that acute exercise has the ability to activate multiple pathways to confer immediate protection against ischaemic events, reduce the severity of potentially lethal ischaemic myocardiac injury, and act as a physiological first line of defence [54]. From an AF-specific perspective, it is possible that the balance between parasympathetic and sympathetic autonomic regulation is involved in the relationship between acute physical (in)activity and AF. For example, AF has been proposed as a disorder of autonomic tone [56] and in ‘athletic AF’, vagal stimulation has an impact on both the atrial refractory period and action potential duration, both of which are key in the development and progression of AF [16]. Nevertheless, in an AF-specific context at least, the mechanisms of any potential acute cardioprotection following short-term or even a single bout of exercise are unknown and warrant future investigation. Other novel work by Linz et al. [57] reported, in 72 patients undergoing pacemaker monitoring for both AF and respiratory disturbances, that nights with more sleep-disordered breathing conferred a higher risk of subsequent AF. Within each patient, nights with the highest sleep apnoea severity conferred a 1.7-fold increase in the risk of having at least 5 min of AF during the same day, compared with the best rated night of sleep. This provides further insight into the acute effects of lifestyle-related risk factors and their potential impact on the heart. This may even partially explain the highly variable patterns seen in paroxysmal AF. In general, AF detection is more likely if we “*look longer, look harder and look with more sophisticated methods….*” [58]. Therefore, more advanced longitudinal monitoring of these dynamic risk factors (e.g., physical activity, sleep quality etc.) may not only help explain the causes of arrythmia but also better inform clinical management and lifestyle interventions beyond traditional risk factors.

## 4. Limitations

Although work in the area of AF-specific exercise rehabilitation is progressing, there are few, relatively small RCTs that have measured AF-specific outcomes such as AF recurrence, AFburden, and AF-specific quality of life. Moreover, detailed and consistent reports of exercise dose (frequency, intensity, time, and type) can be improved to enhance comparison and help determine the most effective interventions for people with AF. There may be differing effects of exercise training across different AF subtypes (paroxysmal, persistent, and permanent); however, there is currently insufficient evidence to meaningfully stratify results in this manner, and most research to date has included mixed AF subtype cohorts. Although we aimed to propose a more nuanced AF-specific exercise rehabilitation recommendation, this is only the first step towards a personalised exercise prescription for people with AF. The latter necessitating individual views and preferences and the use of the most relevant outcomes to those using the intervention, which is an area requiring further research. It is important to note that interventional research in females is limited, with the majority of patients across studies being male. As there are known sex-based differences in terms of physical activity and incident AF (i.e., females may have a higher upper safe threshold compared to males), which we have previously discussed [16]. Whether this is the case for secondary prevention in patients with AF following an intervention warrants further investigation. In the search for the most effective interventions, participant adherence is critical; therefore, reporting intervention adherence and fidelity as part of a high-quality process evaluation would help to determine real-world feasibility and would be integral to ascertain ‘active ingredients’ of rehabilitation programmes.

## 5. Perspectives of Future Directions

Future work in this area should explore whether there are any negative consequences of higher levels of physical activity and exercise training for secondary prevention in patients with AF. In particular, given that at very high levels of vigorous endurance training, such as >5000 MET-minutes/week or 5-to-10 fold the existing physical activity guidelines, we observe an increased prevalence of AF, also known as athletic AF [16]. The exciting prospect of the immediate effects of lifestyle-related risk factors, such as physical activity and sleep quality, warrants further research to understand these dynamic factors and explore strategies to implement such knowledge into personalised treatment strategies. Moreover, suitably powered exercise-based rehabilitation interventions, which include rigorous measures of AF-specific outcomes (e.g., AF burden measured via continuous rhythm monitoring), are needed. The measurement of dynamic risk factors and AF burden is becoming more feasible as new technology and smart devices allow continuous and non-invasive measurement of biomarkers and AF rhythm detection. More research is needed to investigate if there are potentially different therapeutic responses between different AF subtypes. In particular, there is limited research for females and people with permanent AF. Finally, it is central to remember the individual patient in clinical research and the intervention components and outcomes that may be most important to them.

## 6. Conclusions

We propose that a minimum exercise threshold of 360–720 MET-minutes/week, corresponding to 60–120 min exercise per week at moderate-to-vigorous intensity, could be an evidence-based recommendation for patients with AF to improve AF-specific outcomes, quality of life, and possibly long-term major adverse cardiovascular events. This minimum threshold is typical of a cardiac rehabilitation programme, although we would like to emphasise that the dose could be progressively increased(via duration and/or intensity of exercise) up to and perhaps even beyond 1000–1499 MET-minutes/week. This would increase the likelihood of beneficial outcomes and reduce the number of ‘non-responders’. This could be achieved as part of an individualised and progressive rehabilitation programme. In addition, various forms of Yoga seem to have a consistently beneficial effect on the quality of life in patients with AF and would, therefore, seem to be a promising adjunct within an individualised rehabilitation programme.

**Highlights**

- Four systematic reviews and 21 primary studies were included that investigated exercise training or physical activity for people with AF.
- Included studies represented a range of interventions and participant characteristics including females, males, and all AF subtypes.
- We propose 360–720 MET-minutes/week, corresponding to ~60–120 min of moderate-vigorous intensity exercise per week as an evidence-based recommendation for patients with AF.
- This recommendation could be achieved in a variety of ways to suit the individual i.e., two 60 min or four 30 min sessions of moderate-intensity exercise or three 20 min sessions of high-intensity exercise, for example.
- Further work is needed to explore the potential of immediate effects of lifestyle-related risk factors, such as physical activity and sleep quality, on AF recurrence and burden.
- Furthermore, adequately powered RCTs are needed to investigate the effectiveness of exercise-based rehabilitation on AF-specific outcome measures.

## Figures and Tables

**Table 1:** Intervention components/physical activity profile and outcomes of interest from included studies.

First Author, Year	AF Population *n* = Sample; Age (Years); Sex (%); AF Subtype	Exercise Intervention *Frequency; Intensity; Time; Type*	Follow-Up and Impact on Health Outcomes (SAE, Physical Capacity, AF Specific Outcomes, and QoL)
Randomised Controlled Trials			
Luo, 2017 [17]	*n =* 382 63 years 16% female AF and heart failure (excluded if sustained fast AF)	*Frequency*: 3 sessions/week for 36 sessions, followed by transition to a home-based exercise program for 2 years. *Intensity*: NR *Time*: 90 min/week for 3 months, followed by 120 min/week thereafter *Type*: Aerobic exercise (walking, treadmill, or cycle ergometer)	Follow-up: median of 2.6 years SAE: AF was associated with a 24% per year higher rate of mortality/hospitalisation in the control group compared to intervention group (HR: 1.53; 95% CI: 1.34 to 1.74; *p* < 0.001) in unadjusted analysis; this did not remain significant after adjustment (HR: 1.15;95% CI: 0.98 to 1.35; *p* > 0.09). No interaction between AF and exercise training (all *p* > 0.10). No difference in AF event rates between groups (all *p* > 0.10).
Malmo, 2016 [18]	*n =* 51 Intervention: 56 years 77% female Control: 62 years 88% male Paroxysmal or persistent AF	*Frequency*: 3 sessions/week for 12 weeks *Intensity*: Vigorous (85–95% of maximal heart rate and Borg 6–20) *Time*: 4 min intervals *Type*: Walking or running on treadmill	Follow-up: 20 weeks Mean time in AF: 10.4% (95% CI, 4.6–17.8) to 14.6% (95% CI, 6.4–24.9) in the control group vs. 8.1% (95% CI, 4.1–12.8) to 4.8% (95% CI, 2.0–7.6) in the exercise group (*p* = 0.001). V˙O_2_-peak change to follow-Up: Control group: −0.3 ± 4.3 vs. Exercise group: 3.2 ± 2.5, *p* < 0.001. Quality of life change to Follow-Up, SF-36: Physical component score: Control group: −0.3 ± 5.4 vs. exercise group: 2.2 ± 4.4 Mental component score: Control group: 1.4 ± 7.2 vs. Exercise group: 3.6 ± 6.5, *p* > 0.05. No serious adverse events, 2 patients in EG had bursitis episodes, 2 patients in CG had a stroke and ventricular tachycardia.
Osbak, 2011	*n =* 49 Intervention: 70 years Control: 71 years 75% male Permanent AF	*Frequency*: 3 sessions/week for 12 weeks *Intensity*: Vigorous (70% of maximal exercise capacity or Borg scale 14–16/20) *Time*: 1 h *Type*: Group-based aerobic training including ergometer cycling, walking on stairs, running, fitness training on physioballs, and interval training.	Follow-up: 12 weeks Physical capacity: 6MWT: Significantly increased within the intervention group (from 504.4 (85.1) m to 569.9 (92.6) m (*p* < 0.001) and between the groups (EG = 569.9 (92.6) m and CG = 454.1 (95.7), (*p* = 0.001). QoL: MLHF-Q score: intervention group: 21.1 ± 18.0 vs. control group: 15.4 ± 17.5, *p* = 0.03. SF-36 subscales: physical functioning (*p* = 0.02), general health perceptions (*p* = 0.001), and vitality (*p* = 0.02) in favour of the intervention group. No adverse events
Pippa, 2007	*n =* 43 Intervention: 68 years 64% male Control: 68 years 76% male Permanent AF	*Frequency*: 2 sessions/week for 16 weeks *Intensity*: NR (predict light) *Time*: 90 min *Type*: Qigong training (consists of slow and graceful movements with a focus on breathing)	Follow-up: 16 weeks Physical capacity: 6MWT: Intervention group: 531(121) meters at the end of intervention, 474 (109) meters at 16 weeks after intervention vs. control group: 380 (97) meters at the end of intervention, 350 (110) meters after 16 weeks. *p* < 0.001. 1 retinal embolism in the intervention and 1 case of deep vein thrombosis during the follow-up in the control group.
Rienstra, 2018	*n =* 245 Intervention: 64 years 79% male Control: 65 years 79% male Persistent AF	*Frequency*: 2–3 sessions/week for 9–11 weeks *Intensity*: NR *Time*: 20–30 min exercise, *Type*: Cardiac rehabilitation	Follow-up: 1 year AF specific: At 1 year, sinus rhythm was present in 89 (75%) patients in the intervention vs. 79 (63%) in the conventional group (odds ratio 1.765, lower limit of 95% confidence interval 1.021, *p* = 0.042).
Risom, 2016 Long-term follow up: Risom, 2020	*n =* 210 Intervention: 60 years Male 70% Control: 59 years 73% male Paroxysmal or persistent AF	*Frequency*: 3 sessions/week for 12 weeks *Intensity*: Intensity was progressively increased *Time*: 1 h *Type*: Comprehensive cardiac rehabilitation. Cardiovascular training and strength exercises	Follow-up: 6 and 12-months SAE: Mortality: one death in each group at 6- and 24-months follow-up (*p* > 0.99). All hospital admissions: Intervention group: *n* = 71 (68%) vs. control group: *n* = 60 (57%). Physical capacity: V˙O_2_-peak at four months: (Intervention group: 24.3 mL kg^−1^ min^−1^ vs control group: 20.7 mL kg^−1^ min^−1^, *p* = 0.003). VO_2_ peak at twelve months: (Intervention group: 25.8 mL kg^−1^ min^−1^ vs control group: 22.4 mL kg^−1^ min^−1^, *p* = 0.002). OoL: SF-36: General health Perception (Intervention group: 67.16 points vs. control group: 66.9 points. *p* = 0.02, of Interaction between Intervention and time). No significant difference between groups was found at six or 24 at the other domains. AFEQT: The results are in favour of the intervention group: Global score: (Intervention group: 81.64 points vs. control group: 82.87 points *p* = 0.04, of Interaction between Intervention and time) and the treatment satisfaction *p* = 0.03, of Interaction between Intervention and time score at 24 months. Two serious adverse events (AF intervention- related and unrelated to intervention death in the EG and 1 unrelated to intervention death in the CG group. 16 non-serious adverse events EG and 7 in the CG.
Skielboe, 2017	*n =* 76 Low intensity: 64 years 58% male High intensity: 61 years 59% male Paroxysmal or persistent AF	*Frequency*: Two sessions/week for 12 weeks *Intensity*: exercise at either low or high intensity (50% (Borg scale 11–13/20) and 80% (Borg scale 16–18/20) of maximal perceived exertion, respectively) *Time*: 60 min *Type*: 20 min interval exercising on ergometer bike, 20 min varying circuit exercise on the floor.	Follow-up: 16 weeks. SAE: All hospital admissions: 19 patients in each group. Physical capacity: V˙O_2_-peak: No difference between groups: Mean diff. -0.76 mL O2/kg/min, 95% CI -3.22 ± 1.70. AF specific outcomes: Burden of AF measured by daily electrocardiography-reporting for 12 weeks. Results: No statistical difference between low and high intensity exercise for both unadjusted (IRR 0.983, 95% CI 0.39–2.46, *p =* 0.971) and adjusted analyses (IRR 0.742, 95% CI 0.29–1.91, *p =* 0.538). No serious adverse events in both groups. Three unserious adverse events reported in low intensity group and 5 in high intensity group, including symptoms of arrhythmia, hospital admission, AF ahead of an exercise session, and noncardiac complaints.
Wahlström, 2017	*n =* 80 Intervention: 64 years 48% male Control: 63 years 72%male Paroxysmal AF	*Frequency*: One session/week for 12 weeks Intensity: NR (predict light) *Time*: 30 min *Type*: Mediyoga (a therapeutic form of yoga evolved from Kundalini yoga). It is calm, meditative yoga based on deep breathing.	Follow-up: 14 weeks QoL: SF-36, Mental component scale improved in the intervention but not control: Intervention group: baseline 42.1 (17.6–53.5) to follow-up 50.6 (24.0–55.2) points vs. control group: baseline 53.0) 14.7–56.0) to follow-up 52.7 (24.5–57.1) points, *p =* 0.016. Physical component scale no difference within or between groups: Intervention group: 50.2 (27.6–59.1) points vs. control group: 49.0 (29.1–61.6) points, *p =* 0.837.
Joensen, 2019	*n =* 52 Intervention: 62 years 61% male Control: 60 years 71% male Paroxysmal or persistent AF	*Frequency*: 2 sessions/week for 12 weeks *Intensity*: Moderate-to-vigorous intensity (≥ 70% of maximum exercise capacity or 14–16 on the Borg scale) *Time*: 60 min *Type*: Cardiac rehabilitation	Follow-up: 3, 6 and 12-months Physical capacity: Maximum exercise capacity improved in the intervention group from baseline (176 W (SD 48)) to 6 months (190 W (SD 55)). There was no change in the control group. 6MWT was improved in the EG form 613 m (96) to 644 m (84) with no statistically significant differences within or between the groups. AF-QoL-18 significantly improved in the intervention group from 48.4(22.8) to 68.0(15.2) compared with the control group (baseline 51.6 (SD 22.3), 6 months 59.2 (SD 27.3), *p =* 0.031. There was no statistical difference at 12-months. No statistical difference in AFEQT and EQ-VAS between intervention and control. 20 readmissions for cardiac reasons (mostly AF) in the intervention group and 18 in the control group. 13 direct current cardioversions in the intervention group and 12 in the control group. 7 radiofrequency ablations in the intervention group, and 4 in the control group.
Kato, 2019	*n =* 68 Intervention: 67 years 71% male Control: 65 years 90% male Persistent AF	*Frequency*: 1–2 sessions/week, for 24 weeks *Intensity*: Moderate intensity *Time*: 60 min *Type*: Cardiac rehabilitation	Follow-up: 6 months Physical capacity: Significant increases in the 6MWT form 545(123) m to 596 (95) m and also the V˙O_2_-peak from 17.8 (3.4) mL kg^−1^ min^−1^ to 19.8 (4.6) mL kg^−1^ min^−1^ in the intervention group after 6 months with no significant changes in the control group. AF specific: During the six-month follow-up period, 21.4% (6 patients) of the intervention group had AF recurrence and 25.8% (8 patients) in the control group with a risk ratio of 0.83 (95%CI, 0.33 to 2.10). 1 thoracic compression fracture not intervention-related, 1 developed hypothyroidism during the intervention period and no cardiovascular adverse events in either group.
Melo, 2019	*n* = 63 Intervention: 69 years 79% male Control: 66 years 75% male Persistent AF	*Frequency*: 2 sessions/week, for 6 months *Intensity*: Vigorous intensity *Time*: 60 min *Type*: HIIT	Follow-up: 6 months QoL: HQL-14 improved between the groups but with no significant differences. Physical capacity: Significant increases in V˙O_2_-peak after 6 months in both intervention and control; from 12.6 (1.7) mL kg^−1^ min^−1^ to 15.0 (2.3) mL kg^−1^ min^−1^ in intervention and 12.1 (1.7) mL kg^−1^ min^−1^ 15.6 (2.3) mL kg^−1^ min^−1^ in the control.
Wahlström, 2020	n = 152 Intervention 65 years 47% male Active control: 63 years 52% male Control: 64 years 49% male Paroxysmal AF	*Frequency*: 1 session/week, for 12 weeks *Intensity*: NR (predict light) *Time*: 60 min/session *Type*: Yoga	Follow-up: 12 weeks QoL: SF-36 significantly improved within Medi-yoga intervention group in the Bodily Pain from 70 (27) to 83 (19), (*p =* 0.014), General Health from 61 (18) to 70 (17), (*p =* 0.037), Social Function from 75 (28) to 88 (18), (*p =* 0.029), Mental Health from 64 (16) to 72 (16), (*p =* 0.030) and Mental Component Summary score from 40 (11) to 46 (9), (*p =* 0.019), subscales, however, no significance between group effects. No adverse events.
**Cohort studies**			
Ahn, 2021	*n =* 66,692 60 years 64% male Newly diagnosed AF	Cohort stratified by: Persistent non-exercisers (30.5%) New exercisers (17.8%), Exercise dropouts (17.4%) Exercise maintainers (34.2%) *Exercise “Yes” = performing moderate (>30 min) or vigorous intensity exercise (>20 min), at least once a week. Exercise “No” = not engaging in any moderate or vigorous intensity exercise. Therefore, persistent non-exercisers (No to No), new exercisers (No to Yes), exercise dropouts (Yes to No), and exercise maintainers (Yes* to Yes).	Follow-up: 2 years SAE: The new exerciser and exercise maintainer groups were associated with a lower risk of HF compared to the persistent non-exerciser group: the hazard ratios (HRs) (95% Cis) were 0.95 (0.90–0.99) and 0.92 (0.88–0.96), respectively (*p <* 0.001). Performing exercise any time before or after AF diagnosis was associated with a lower risk of mortality compared to persistent non-exercising: the HR (95% CI) was 0.82 (0.73–0.91) for new exercisers, 0.83 (0.74–0.93) for exercise dropouts, and 0.61 (0.55–0.67) for exercise maintainers (*p <* 0.001). For ischemic stroke, HRs were 10%–14% lower in patients of the exercise groups, yet differences were statistically insignificant (*p =* 0.057). Energy expenditure of 1000–1499 MET-min/wk (regular moderate exercise 170–240 min/wk) was consistently associated with a lower risk of each outcome based on a subgroup analysis of the new exerciser group.
Bonnesen, 2021	*n* = 1410 75 years 46% female Participants had AF risk factors but no prior AF diagnosis	A dynamic parameter describing within-individual changes in daily physical activity, i.e., average daily activity in the last week compared to the previous 100 days, was computed and used to model the onset of AF.	Follow-up: 3.5 years A 1 h decrease in average daily physical activity was associated with AF onset the next day (odds ratio 1.24 (1.18–1.31)). This effect was modified by overall level of activity (*p* < 0.001 for interaction), and the signal was strongest in the tertile of participants with lowest activity overall (low: 1.62 (1.41–1.86), mid: 1.27 (1.16–1.39), and high: 1.10 (1.01–1.19)).
Buckley, 2021a	*n* = 23,894 68 years 30% female Included paroxysmal, persistent and permanent AF	Comprehensive cardiac rehabilitation programme *	Follow-up: 18 months Exercise-based CR was associated with 68% lower odds of all-cause mortality (odds ratio, 0.32; 95% CI, 0.29–0.35), 44% lower odds of rehospitalisation (0.56; 95% CI, 0.53–0.59), and 16% lower odds of incident stroke (0.84; 95% CI, 0.72–0.99) compared with propensity-score matched controls. The beneficial association of exercise-based CR on all-cause mortality was independent of sex, older age, comorbidities, and AF subtype.
Buckley, 2021b	*n* = 9808 70 years 32% female Included paroxysmal AF	Comprehensive cardiac rehabilitation programme *	Follow-up: 2 years Progression from paroxysmal AF to sustained AF (persistent/permanent) at 2-year follow-up was proportionally lower with 19.3% (*n* = 617 of 3197 patients) in the exercise-based CR cohort compared to 24.5% (*n* = 909 of 3716 patients) in the matched controls (OR 0.74, 95% CI: 0.66–0.83).
Garnvik, 2020	*n =* 1117 Inactive: 73 years 61% male Not meeting: 71 years 67% male Meeting: 69 years 78% male All AF subtypes	Cohort stratified into 3 groups: (1). Inactive, reflecting no PA or less than once a week. (2). Below guideline amount, reflecting < 150 min of moderate intensity or <75 min of vigorous intensity/week. (3). At or above guideline amount, ≥150 min moderate or ≥75 min vigorous intensity/week.	Follow-up: up to 9 years or an event Atrial fibrillation patients meeting PA guidelines had lower risk of all-cause (hazard ratio (HR) 0.55, 95% confidence interval (CI) 0.41–0.75) and CVD mortality (HR 0.54, 95% CI 0.34–0.86) compared with inactive patients. The respective HRs for CVD morbidity and stroke were 0.78 (95% CI 0.58–1.04) and 0.70 (95% CI 0.42–1.15). Each 1-MET higher eCRF was associated with a lower risk of all-cause (HR 0.88, 95% CI 0.81–0.95), CVD mortality (HR 0.85, 95% CI 0.76–0.95), and morbidity (HR 0.88, 95% CI 0.82–0.95).
Hegbom, 2006 (Originally an RCT, but results presented as a single arm pre-post study)	*n =* 30 62 years 88% male Permanent AF	*Frequency*: 3 sessions/week for 8 weeks *Intensity*: 70–90% of maximum heart rate *Time*: 1.25 h *Type*: Three 15 min periods of aerobics at HRmax, interrupted by strengthening exercise for the back, thighs, and stomach 15 min of stretching and relaxation	Follow-up: 8 weeks Physical capacity: Increase in cumulated work at Borg-17 scale: The intervention group (39% ± 38%) and control group (42% ± 35%). Quality of life change to follow-up, SF-36 (within intervention group): from 49 ± 6 pre-training to 52 ± 6 post-training (*p* < 0.05).
Lakkireddy, 2013	*n =* 49 61 years 47% male Paroxysmal AF	*Frequency*: 2 sessions/week for 12 weeks *Intensity*: NR (predict light) *Time*: NR *Type*: Yoga	Follow-up: 12 weeks AF specific: Yoga training reduced symptomatic AF episodes (3.8 ± 3 vs. 2.1 ± 2.6, *p* < 0.001), symptomatic non-AF episodes (2.9 ± 3.4 vs. 1.4 ± 2.0; *p* < 0.001), asymptomatic AF episodes (0.12 ± 0.44 vs. 0.04 ± 0.20; *p* < 0.001). QoL: SF-36 significantly improved after the intervention period (Physical Functioning from 85.0 (80.0–95.0) to 90.0 (85.0–95.0), (*p =* 0.017), General Health from 65.0 (50.0–77.5) to 75.0 (65.0–82.5), (*p <* 0.001), Vitality from 84.0 (68.0–88.0) to 91.0 (80.0–95.8), (*p <* 0.001), Social Functioning from 100.0 (75.0–100.0) to 100.0 (90.0–100.0), (*p =* 0.019), and Mental Health from 75.0 (65.0–85.0) to 80.0 (70.0–86.0), (*p <* 0.001). No adverse events.
Younis, 2018	*n = 304 (pre-post AF arm)* 68 years 76% male AF subtype NR	*Frequency*: 2 sessions/week for 6 months *Intensity*: NR *Time*: 60 min *Type*: Cardiac rehabilitation	Follow-up: 6-months Physical capacity: Significant improvement (delta > 5%) was achieved among 194 (64%) patients with AF.

AFEQT, atrial fibrillation quality of life survey; AF, atrial fibrillation; AF-QoL-18, quality of life questionnaire for patients with atrial fibrillation; MET, metabolic equivalent (one MET = 3.5 mL.g.min of oxygen consumption); RCT, randomised controlled trial; SAE, serious adverse event; SF-36, 36-item short form survey; QoL, quality of Life; 6MWT, 6 min walk test. * Individual exercise programme details unknown due to clustering of data from multiple sites using a network research database.
